# Morphological canalization, integration, and plasticity in response to population density in *Abutilon theophrasti*: Influences of soil conditions and growth stages

**DOI:** 10.1002/ece3.7960

**Published:** 2021-07-28

**Authors:** Shu Wang, Dao‐Wei Zhou

**Affiliations:** ^1^ College of Forestry Forest Ecology Research Center Guizhou University Guiyang China; ^2^ Northeast Institute of Geography and Agroecology Chinese Academy of Sciences Changchun China

**Keywords:** competition intensity, developmental canalization, growth stage, phenotypic integration, phenotypic plasticity, soil conditions

## Abstract

Phenotypic integration and developmental canalization have been hypothesized to constrain the degree of phenotypic plasticity, but little evidence exists, probably due to the lack of studies on the relationships among the three processes, especially for plants under different environments. We conducted a field experiment by subjecting plants of *Abutilon theophrasti* to three densities, under infertile and fertile soil conditions, and analyzing correlations among canalization, integration, and plasticity in a variety of measured morphological traits after 50 and 70 days, to investigate the relationships among the three variables in response to density and how these responses vary with soil conditions and growth stages. Results showed trait canalization decreased and phenotypic integration and the degree of plasticity (absolute plasticity) in traits increased with density. Phenotypic integration often positively correlated with absolute plasticity, whereas correlations between trait canalization and plasticity were insignificant in most cases, with a few positive ones between canalization and absolute plasticity at low and medium densities. As plants grew, these correlations intensified in infertile soil and attenuated in fertile soil. Our findings suggested the complexity of the relationship between canalization and plasticity: Decreased canalization is more likely to facilitate active plastic responses under more favorable conditions, whereas increased level of integration should mainly be an outcome of plastic responses. Soil conditions and growth stage may affect responses of these correlations to density via modifying plant size, competition strength, and plastic responses in traits. We also predicted that decreased canalization can be advantageous or disadvantageous, and the lack of response to stress may demonstrate a stronger ability of adaptation than passive response, thus should be adaptive plasticity as active response.

## Graphical abstract

The positive correlations between integration and the degree of plasticity in traits suggested that increased phenotypic integration may not constrain the plastic responses of plants to environments, but rather a consequence of such responses.

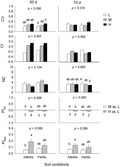

## 1 INTRODUCTION

Phenotypic variations due to environmental or developmental disturbances are very ubiquitous. Plants are able to react to or buffer against these disturbances through multiple processes, such as phenotypic plasticity, developmental canalization, phenotypic integration, and developmental stability (Debat & David, 2001). Different processes may have opposite effects, which may counteract each other, producing relatively variable or stable phenotypes ultimately. Consequently, the extent of phenotypic variation expressed in traits may largely depend on the relationships or co‐operations between these processes.

Phenotypic plasticity, defined as the ability to produce different phenotypes in response to different environmental conditions (Bradshaw, 1965; Pigliucci, 2005), might be a process leading to increased phenotypic variation. Not all species or traits are plastic, probably because the production of plasticity is limited by both intrinsic and extrinsic factors (DeWitt et al., 1998; Givnish, 2002; Valladares et al., 2007). For example, developmental canalization (or robustness), which indicates the property of an organism that buffers development against environmental and genetic perturbations to produce a consistent phenotype (Waddington, 1957), reflects an effort to reduce phenotypic variation. This is especially obvious when developmental canalization is classified into environmental canalization and genetic canalization (Wagner et al., 1997), and environmental canalization, defined as the insensitivity of traits to external perturbations in variable environments (Debat & David, 2001; Stearns et al., 1995; Wagner et al., 1997), is actually a process in opposition to phenotypic plasticity (Stearns et al., 1995; Wilkins, 1997). However, both phenotypic plasticity and developmental canalization are the ability of an organism to adjust phenotypic expression appropriately in dealing with environmental changes, at individual and population levels, respectively (Reed et al., 2010; Schlichting & Pigliucci, 1998). As stress conditions generally increase phenotypic variability in traits (Woods et al., 1999), if both plasticity and instability (contrary to canalization and developmental stability) of traits increase with environmental stress, it is reasonable to infer some common mechanisms underlying the two processes (Meiklejohn & Hartl, 2002). However, direct evidence is scarce on the relationships between trait plasticity and canalization, even less on their variations with environments, especially in plant species.

On the other side, the evolution of a given trait and its plasticity may also be restricted by its genetic correlations with other traits (Agrawal & Stinchcombe, 2009; Gianoli & Palacio‐López, 2009). The expression of a phenotype under a certain environment is an integrative result of all local responses of many modular traits and their interactions (Pigliucci & Preston, 2004). Phenotypic integration, defined as the pattern and magnitude of character correlations due to genetic, developmental, and/or functional connections among traits (Pigliucci & Preston, 2004; Schlichting & Pigliucci, 1998), might be another internal constraint to phenotypic plasticity (Gianoli, 2001, 2003; Pigliucci et al., 1995; Schlichting, 1986, 1989, 1989,1986, 1989, 1989; Valladares et al., 2007). This has been supported by that the degree of plasticity in response to shading or drought in a given trait decreased with the increase of the number of its correlations with other traits, in two local species from Chile (Gianoli & Palacio‐López, 2009). However, the strength of phenotypic integration can increase with environmental stresses (García‐Verdugo et al., 2009; Gianoli, 2004; Schlichting, 1989, 1989,1989, 1989; Waitt & Levin, 1993). Meanwhile, environmental stress also induce plastic responses in traits. It suggested a positive relationship or co‐operation between phenotypic integration and plasticity. The contradicted hypotheses entail studies on their relationships under different environments, whereas most empirical studies have focused on patterns of phenotypic integration in a single environment only (Pigliucci & Preston, 2004; but see Liu et al., 2007; Pigliucci et al., 1995); we know very little about how phenotypic integration and its relationship with plasticity may vary with environmental conditions (Mallitt et al., 2010).

Both variation among individuals and phenotypic plasticity can contribute to integration among morphological traits (Klingenberg, 2014), leading to possible correlations among them. Biotic and abiotic conditions and growth stages may affect these correlations, through effects on plant size and trait plasticity (Wang et al., 2017). Relevant studies including different stages of plant growth and/or under different biotic or abiotic conditions are needed, to generalize about the relationships among the three processes (Kavanagh, 2020). The increase of population density, as one of major natural biotic stresses, can result in heterogeneity in multiple environmental factors, inducing complex plasticity in traits (Wang et al., 2017, 2021). It is unknown whether and how plasticity will correlate with developmental canalization and/or phenotypic integration in response to density and effects of soil conditions and plant growth stage.

To investigate the relationships between canalization, integration, and phenotypic plasticity in response to density, and their variations with abiotic environments and growth stages, we conducted a field experiment with an annual herbaceous species of *Abutilon theophrasti*, by subjecting plants to three densities under two contrasting soil conditions, to measure a number of morphological traits and analyze correlations among plasticity, canalization, and integration in these traits, at two stages of plant growth. We aimed to test the following hypotheses: (1) developmental canalization decreases and integration and plasticity in traits increase, with higher densities; (2) there are certain correlations among the three processes, which intensify with greater densities; and (3) soil conditions and growth stage can influence responses of these correlations to density.

## 2 MATERIALS AND METHODS

### 2.1 Study species

*Abutilon theophrasti* Medicus (Malvaceae) is an annual weedy species, native to China and India. It usually grows to a height of up to 1–1.5 m and can reach reproductive maturity within 90 days, completing its life cycles in ~5 months (McConnaughay & Coleman, 1999), with substantial plasticity in allocation, morphology, and architecture in response to varying environmental factors (McConnaughay & Bazzaz, 1992). It colonizes relatively nutrient‐rich habitats and is typically found in open fields, on roadsides, and in gardens.

### 2.2 Experimental design

The experiment was conducted between June and August in 2007 at the Pasture Ecological Research Station of Northeast Normal University, Changling, Jilin province, China (44°45’ N, 123°45’ E). The environmental conditions for plant growth were very close to natural. Seeds of *A. therophrasti* were collected from local wild populations near the research station in the late August of 2006 and were dry stored at −4°C. We used a split‐plot design, with soil conditions as the main factor, and density and block as a subfactor. Two large plots were assigned as two (infertile and fertile) soil conditions, each was divided into nine 2 × 3 m subplots and randomly arranged with three treatments of densities and blocks. Seeds of *A. theophrasti* were sown on June 7, 2007, with three interplanting distances of 30, 20, and 10 cm, to reach target plant densities of 13.4, 36, and 121 plants/m^2^, assigned as relatively low‐, medium‐, and high‐density treatments, respectively. Most seeds emerged 4 days after sowing. Seedlings were thinned to the target densities at four‐leaf stage. Plots were hand‐weeded when necessary and watered regularly.

We established the infertile soil conditions as a plot using the original soil of experimental field at the station that had been used annually for many years (aeolian sandy soil). The fertile soil conditions were set up by covering the other large plot with 5–10 cm virgin soil transported from a nearby meadow with no cultivation history (meadow soil), with contrasting nutrient contents of the two soil conditions (Wang et al., 2017). The meadow soil is not located far away from the experiment field, which used to be meadow as well and has been reclaimed for experimental use since the establishment of research station. Therefore, basically the soil of the experimental field was the same type as the meadow soil, but with different conditions or qualities. Covering the other plot with meadow soil led to a greater amount of soil or nutrients for the fertile soil treatment, which also led to thicker soil layers of the fertile plot than the infertile one. To keep the soil and resource amounts as even as possible, we crushed the blocky soil into very small bits and mingled them adequately, before spreading them over the entire plot and compaction. Seeds were sown into all plots at the same burial depth and sowing rate.

### 2.3 Data collection

Plants were harvested at 50 and 70 days of plant growth, representing developmental stages of early vegetative growth, late vegetative or early reproductive growth, and middle‐to‐late reproductive growth, respectively. At each stage, six individual plants were randomly chosen from each plot, making a total of 6 replicates ×3 plots ×3 densities ×2 soils ×2 stages = 216 samplings. For each plant, the following traits were measured if applicable: the length of stem, diameter at the basal of stem, petiole length and angle, leaf number, lamina width (lamina size), branch length, angle and number, main root length, main root diameter, lateral root length, and lateral root number (above or equal to 1 mm in diameter along the main root). Morphological traits of plants at 30 days of growth were not taken into account due to small plant sizes. Each individual plant was then separated into root, stem, petiole, leaf, reproductive, and branch parts if any, oven‐dried at 75°C for two days and weighed.

### 2.4 Statistical analysis

All statistical analyses were conducted using SAS statistical software (SAS Institute 9.0 Inc. 2002). All traits were used in analyses (abbreviations see Table 1). All data were log‐transformed except for petiole angles and branch angles (square root‐transformed) to minimize variance heterogeneity before statistical analysis. Three‐way ANOVA and ANCOVA were performed to evaluate the overall effects of growth stage, soil condition, and population density and their interactions on all traits, with total mass nested in growth stage as a covariate in three‐way ANCOVA. Within each soil condition at each stage, effects of density were analyzed by one‐way ANOVA for total mass and one‐way ANCOVA for all the other traits with total mass as a covariate. Adjusted mean values of traits were produced from multiple comparisons by least significant difference (LSD) method of the general linear model (GLM) program in ANCOVAs and were used in calculation of plasticity.

**TABLE 1:** All traits and variables with abbreviations and transformations in this study

Trait/variable	Unit	Abbreviation	Transformation
Total mass	g	TM	Log
Root mass ratio	/	RMR	Log
Stem mass ratio	/	SMR	Log
Petiole mass ratio	/	PMR	Log
Lamina mass ratio	/	LAMR	Log
Reproductive mass ratio	/	REMR	Log
Stem length	cm	SL	Log
Stem diameter	mm	*SD*	Log
Main root length	cm	MRL	Log
Main root diameter	mm	MRD	Log
Lateral root length	cm	LRL	Log
Lateral root number	/	LRN	Log
Petiole length	cm	PL	Log
Petiole angle	^o^	PA	Sqrt
The number of leaves	/	LN	Log
Lamina size	mm	LS	Log
Coefficient of variation	/	CV	Log
Coefficient of integration	/	CI	/
Number of significant correlations	/	NC	Log
Relative plasticity	/	PI_rel_	/
Absolute plasticity	/	PI_abs_	/

The plasticity in traits was evaluated with the revised simplified relative distance plasticity index (RDPI_s_), for its strong statistical power in tests of differences in plasticity (Valladares et al., 2006). We abbreviated RDPI_s_ to PI and calculated it in a given trait in response to high and medium versus low densities (H‐L PI and M‐L PI) as:

PI=(X‐Y)/(X+Y)

where *X* was the adjusted mean trait value at high or medium density, and Y was the mean value at low density. Both level and degree of plasticity (relative plasticity and absolute plasticity) in traits were calculated as PI_rel_ and PI_abs_, respectively.

Phenotypic canalization was evaluated by coefficient of variation (CV) for a given trait, calculated as the standard deviation divided by mean value of the trait. Phenotypic integration was estimated with the number of significant correlations of a trait with other traits (NC; *p* < .05) and coefficient of integration (CI; Cheverud et al., 1983). Correlations among traits were evaluated by Pearson correlation coefficients (PCC) produced by PROC CORR (Gianoli & Palacio‐López, 2009). CI for traits was computed as:

$$I={\left[\sum {\left(\lambda ‐1\right)}^{2}/\left(n^{2}‐n\right)\right]}^{1/2}$$

where n is the number of traits and *λ* is an eigenvalue of the correlation matrix of the normalized data.

Both correlation and regression analyses were applied to qualify and quantify the relationships between phenotypic plasticity (PI) and phenotypic canalization (CV) or integration (NC) at different densities for plants in each soil conditions at each stage. Results of correlations and regressions were also analyzed with three‐way ANOVA to access effects of population density, soil conditions, and growth stage and their interactions; and one‐way ANOVA for effects of density on these relationships in each soil conditions at each stage.

## 3 RESULTS

### 3.1 Responses of variables to density

**FIGURE 1:**
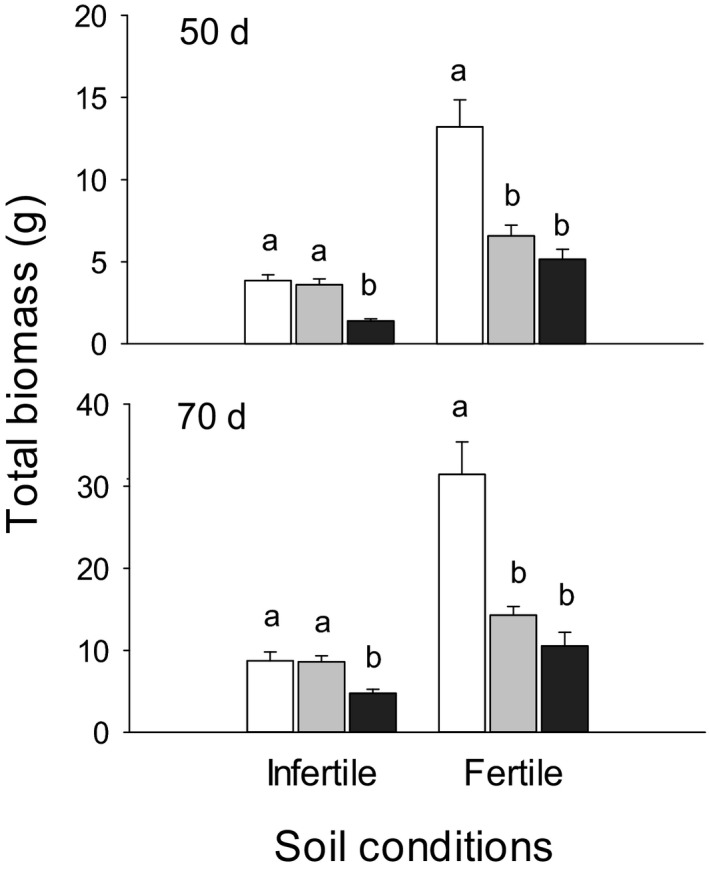
Mean values (±SE) of total biomass for individual plants at low (white), medium (gray), and high (black) densities in infertile and fertile soil conditions at 50 and 70 days of plant growth. Different letters denote differences between density treatments within each soil conditions (*p* < .05)

**FIGURE 2:**
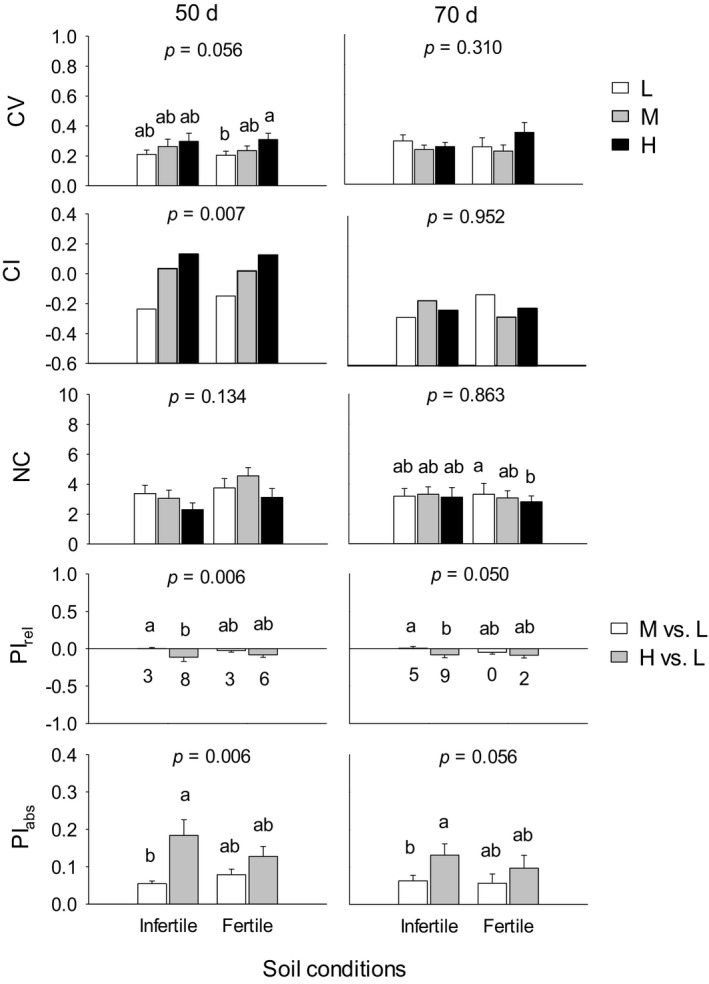
Mean values of coefficient of variation (CV), coefficient of integration (CI), and mean number of significant correlations with other traits (NC) for all traits at low (L), medium (M), and high (H) densities, and mean relative plasticity (PI_rel_) and absolute plasticity (PI_abs_) of all traits in response to medium (M versus L) and high (H versus L) relative to low densities, for plants under infertile and fertile soil conditions at 50 and 70 days of growth. The numbers below the bars in the fourth row represent the numbers of traits that expressed significant degree of plasticity. Different letters denote significant differences between densities and soil conditions; *p* values denote significant differences between densities across two soil conditions (*p* < .05)

Across both stages, infertile versus fertile soil, and high, medium versus low density reduced total biomass (*p* < .001; Figure 1). Although plant size (total mass) explained significant variations in most traits, effects of stage, soil, density, and interaction between stage and soil were still significant for most traits, and interactions between stage and density, between soil and density were also significant for several different traits (Table A1). Density had significant effects on mean coefficient of variation (CV), mean number of correlations (NC), and mean degree of plasticity (PI_abs_) for all traits (*p* < .05; Table 2) and responses of these variables to density varied with soil conditions and growth stages (Figure 2; Tables A2, A3 and A2, A3). Compared to low density, high and medium densities increased CI (coefficient of integration) of traits across both soil conditions at 50 days (LSD, *p* = .007), but not at 70 days; high density increased mean CV by 20% at 50 days (*p* = .046) and decreased mean NC slightly at 70 days (*p* = .067) in fertile soil. Across both growth stages, mean PI_abs_ in response to high versus low density was greater than that in response to medium versus low density in infertile soil (*p* < .001; Figure 2). Total mass was highly plastic, with the average relative plasticity (PI_rel_) of −0.433 in response to high density across all soil conditions and stages, while PIs of other traits varied with soil conditions and growth stages (Table A4).

**TABLE 2:** *F*‐values for three‐way ANOVAs on mean values of relative plasticity (PI_rel_), absolute plasticity (PI_abs_), log‐transformed number of significant correlations among traits (NC) and coefficient of variation (CV) for all traits, showing effects of growth stage (GS), soil condition (SC), population density (PD), and their interactions

Source of Variation	PI_rel_		PI_abs_		Log (NC)		Log (CV)	
	*F*	*p*	*F*	*p*	*F*	*p*	*F*	*p*
GS	0.08	.799	0.95	.337	0.21	.645	0.00	.957
SC	0.07	.775	0.39	.539	0.58	.448	0.26	.612
PD	2.44	.129	**5.26**	.**029**	**3.40**	.**019**	**3.51**	.**032**
GS × SC	2.12	.155	3.55	.069	0.07	.792	0.11	.739
GS × PD	0.87	.359	0.72	.402	2.37	.072	1.00	.369
SC × PD	0.00	.958	0.93	.341	0.56	.640	1.04	.355
GS × SC × PD	0.62	.437	1.27	.268	1.10	.351	0.41	.664

Note

Bold fonts indicate significant effects (*p* < .05).

### 3.2 Relationships among variables

**FIGURE 3:**
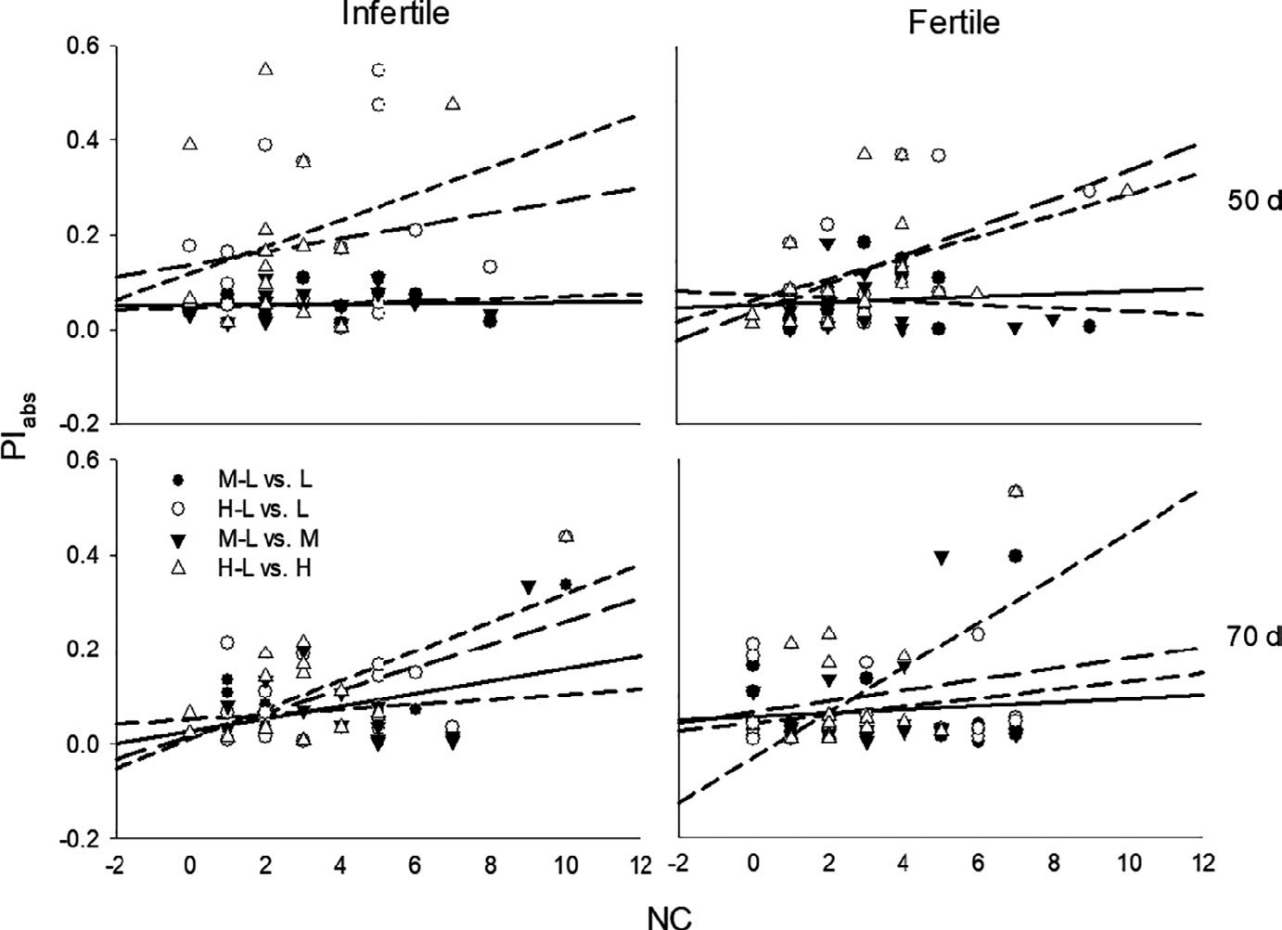
Regressions on the degree of plasticity (PI_abs_) in response to medium (M‐L, black shapes with solid line and long dash line) and high (H‐L, white shapes with long dash line and short dash line) relative to low densities, against the number of significant trait correlations (NC) at low (L, circles with solid line and long dash line), medium (M, black triangle with medium dash line), and high (H, white triangle with short dash line) densities, for plants under infertile and fertile soil conditions at 50 and 70 days of growth

**FIGURE 4:**
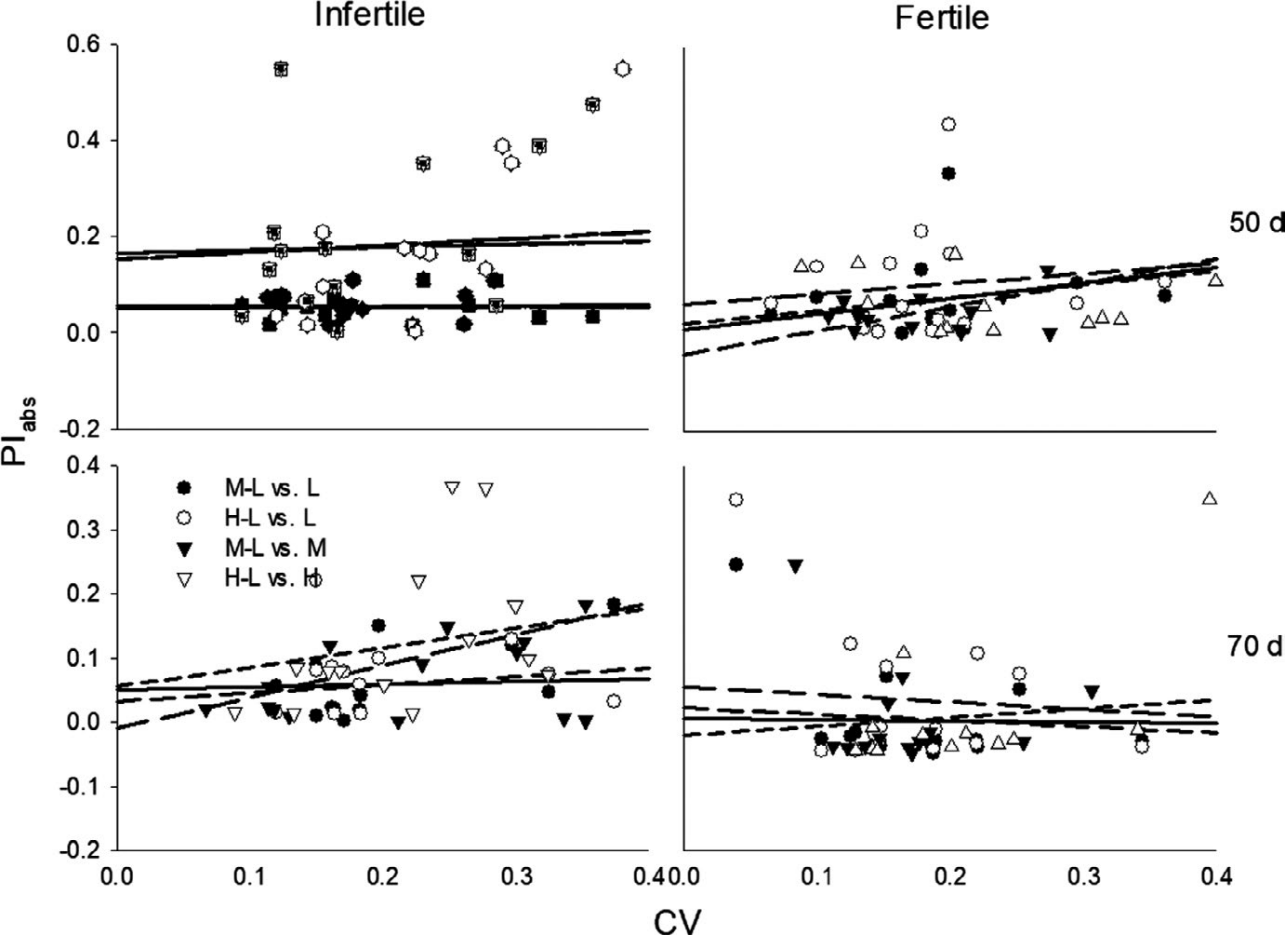
Regressions on the degree of plasticity (PI_abs_) in response to medium (M‐L, black circle with solid line and triangle with long dash line) and high (H‐L, white circle with long dash line and triangle with short dash line) relative to low densities, against coefficient of variation (CV) at low (L, circle with solid line and long dash line), medium (M, black triangle with medium dash line), and high (H, white triangle with short dash line) densities, under infertile and fertile soil conditions, for plants at 50 and 70 days of growth

Effects of soil conditions, growth stage, and population density and their interactions were more significant for correlations between NC and PI_abs_, and effects of growth stage and population density were more significant for correlations between CV and PI_rel_, than other correlations (Table 3). Trait NC negatively correlated with PI_rel_ and positively correlated with PI_abs_, but little correlation was found between trait CV and PI, except for a few positive correlations between CV and PI_abs_ at low and medium densities; and these correlations decreased over time in fertile soil and increased over time in infertile soil (Table 4; Figures 3 and 4). For both stages, density had more significant effects on the slopes and intercepts for relationships between PI and NC in fertile soil conditions than in infertile soil, whereas little difference due to density was found for correlations between PI_abs_ and CV (Table 5; Figures 3 and 4). At 50 days, the slopes for relationships between PI_abs_ of plasticity to high versus low density (H‐L PI_abs_) and NC at high and low densities were 0.038 and 0.031, respectively, significantly higher than the 0.004 and 0.006 for the relationships between plasticity to medium versus low density (M‐L PI_abs_) and NC at medium and low densities (*p* < .001); at 70 days, the slope for the relationship between H‐L PI_abs_ and NC at high density was 0.051, 740% higher than the mean values of the other slopes (0.006; *p* = .001; Figure 3).

**TABLE 3:** *F*‐values for three‐way ANOVAs on the (Partial) Pearson Correlation Coefficients for relationships among plasticity (relative plasticity [PI_rel_] and absolute plasticity [PI_abs_]), number of significant correlations among traits (NC), and coefficient of variation (CV), showing effects of soil condition (SC), growth stage (GS), population density (PD), plasticity type (PT), and their interactions

Source of variation	PI_rel_		PI_abs_	
	NC	CV	NC	CV
SC	4.11	0.70	61.52**	8.54*
GS	1.30	14.49*	23.90**	0.62
PD	5.47	18.36**	105.29***	2.10
PT	1.10	18.02*	191.06***	0.06
SC × GS	2.97	31.77**	272.81***	54.98**
SC × PD	0.42	35.24**	173.68***	2.84
SC × PY	3.32	50.33**	270.46***	14.06*
GS × PD	0.56	1.92	4.05	2.47
GS × PT	0.71	0.56	33.92**	8.88*
PD × PT	0.36	6.71*	4.90	1.70
SC × GS × PD	0.32	7.97*	75.35***	3.44
SC × GS × PT	0.01	0.06	82.38***	1.17
SC × PD × PT	0.02	2.41	26.25**	1.46
GS × PD × PT	0.55	2.39	12.22*	0.85

Note

*p < .10, **p < .05, ***p < .01.

**TABLE 4:** Correlations of trait plasticity (PI) with the number of significant correlations among traits (NC) and coefficient of variation in traits (CV) at each density and across all densities under infertile and fertile soil conditions at 50 and 70 days of plant growth

Stage	PI	NC			CV		
		Low	Medium	High	Low	Medium	High
50 days							
Infertile	H‐L PI_rel_	0.013	–	−0.318	−0.153	–	−0.198
	M‐L PI_rel_	−0.112	−0.107	–	0.190	0.196	–
	H‐L PI_abs_	0.042	–	−0.223	0.095	–	0.079
	M‐L PI_abs_	0.179	0.264	–	0.067	−0.033	–
Fertile	H‐L PI_rel_	−0.470*	–	−0.610**	−0.097	–	−0.417
	M‐L PI_rel_	−0.405	−0.208	–	−0.170	−0.359	–
	H‐L PI_abs_	0.382	–	0.538**	0.216	–	0.412
	M‐L PI_abs_	0.556**	0.200	–	0.414	0.699***	–
70 days							
Infertile	H‐L PI_rel_	0.027	–	−0.107	−0.236	–	−0.101
	M‐L PI_rel_	−0.462*	−0.271	–	−0.111	0.118	–
	H‐L PI_abs_	0.099	–	−0.122	0.682***	–	0.288
	M‐L PI_abs_	0.507**	0.449*	–	0.126	0.262	–
Fertile	H‐L PI_rel_	−0.153	–	−0.640***	0.353	–	−0.409
	M‐L PI_rel_	−0.297	−0.269	–	0.021	0.154	–
	H‐L PI_abs_	0.110	–	0.675***	−0.274	–	0.364
	M‐L PI_abs_	0.243	0.202	–	−0.063	−0.211	–

Note

Abbreviations for all variables are in Table 1.

*p < .10, **p < .05, ***p < .01.

**TABLE 5:** *F*‐values for GLM on density effects on regressive relationships between relative plasticity (PI_rel_) and the degree of plasticity (absolute plasticity, PI_abs_) and the number of significant correlations among traits (NC), coefficient of variation (CV) for all traits, under infertile and fertile soil conditions at 50 and 70 days of plant growth

Plasticity	Stage (days)	Soil conditions	NC		CV	
			*F*_Slope_	*F*_Intercept_	*F*_Slope_	*F*_Intercept_
PI_rel_	50	Infertile	0.41	0.70	0.46	2.48*
		Fertile	11.97***	0.69	0.20	0.15
	70	Infertile	1.39	2.17*	0.29	2.30*
		Fertile	6.30***	4.18***	0.66	0.54
PI_abs_	50	Infertile	0.92	0.69	0.08	5.89***
		Fertile	6.29***	1.35	0.39	0.63
	70	Infertile	0.56	0.61	2.06	3.16**
		Fertile	5.93***	4.61***	0.66	0.65

Note

Significance levels: *p < .10, ** *p* < .05, ***p < .01.

## 4 DISCUSSION

### 4.1 Developmental canalization in response to density

Comparing the among‐individual variations (CV) of different traits, we found CV of reproductive mass ratio was higher than other traits at different densities, consistent with other study (Woods et al., 1999), and reproductive mass ratio did not show lower plasticity than other traits either. These suggested the relative stability of fitness traits depends on the level or range of environmental variations, and increased phenotypic variations can be produced under highly stressful conditions that plants are incapable to adapt (Woods et al., 1999). It is theoretically predicted that morphological traits tend to have relatively higher levels of plasticity under increasing environmental variations (Bradshaw, 1965; de Jong, 1995); by contrast, fitness traits may be more likely to maintain stable under a range of environmental conditions (Lerner, 1954; Stearns & Kawecki, 1994; Waddington, 1957; Wagner et al., 1997). Because characters more closely related to fitness are expected to be better buffered against environmental effects, deviations from the optimal phenotype will be strongly selected against (Clarke, 1995; Lerner, 1954; Stearns & Kawecki, 1994; Waddington, 1957). These predictions have been proved by the contrasting performances in the degree of plasticity in response to temperature between morphological traits and fitness traits (Liefting et al., 2009). However, when the stress is severe enough, the buffering against drastic changes may no longer be able to prevent such overt changes, and phenotypic variability in more robust traits might assist survival at the population level (Elgart et al., 2015). The increase of density should have been severe enough to cause the decrease in reproductive allocation (passive plasticity), leading to decreased canalization, increased phenotypic integration, and stronger correlations among these variables.

### 4.2 Phenotypic integration in response to density

In spite of the recognized importance that changes in the correlation structure can have for evolutionary change (Lande & Arnold, 1983), we still know surprisingly little about how the environment influences levels of phenotypic integration. Despite much recent progress on this topic (Pigliucci & Preston, 2004; Schlosser & Wagner, 2004), most empirical studies have only studied patterns of phenotypic integration in a single environment (Pigliucci & Preston, 2004); but see (Liu et al., 2007; Pigliucci et al., 1995). In this study, we found an increase in coefficient of integration (CI) with density at 50 days, consistent with some studies (Gianoli, 2004; Schlichting, 1986; Wylde & Bonduriansky, 2020), suggesting the strength of phenotypic integration can increase with environmental stresses (García‐Verdugo et al., 2009; Gianoli, 2004; Schlichting, 1989, 1989,1989, 1989; Waitt & Levin, 1993). The increase in the number and strength of correlations among functionally correlated traits (phenotypic integration) is related to the extent of environmental stress (Gianoli, 2004; Schlichting, 1986) and endow plants the ability to effectively respond to such stress (Chapin III, 1991). Because environmental stress can trigger certain mechanisms of morphological responses, inducing greater variations in traits. In the absence of environmental stress, different modular traits function by themselves, with little relation to each other; in the presence of stress, changes in some traits may drive the changes of other traits, leading to stronger correlations among traits. This accorded with the increase in responses of mean trait values (PI) and coefficient of variation (CV) with increased density. Increased density led to reduced plant size, and passive plasticity, decreased canalization, and increased integration should all be outcomes of adverse effects of increased density. The decrease in the number of correlations (NC) with density occurred in fertile soil at 70 days only, consistent with other studies (Badyaev et al., 2005; Mallitt et al., 2010; Pigliucci & Kolodynska, 2002), suggesting attenuated strength of density effects over time (Wang et al., 2017), due to small individuals being obsoleted.

### 4.3 Phenotypic plasticity in response to density

**FIGURE 5:**
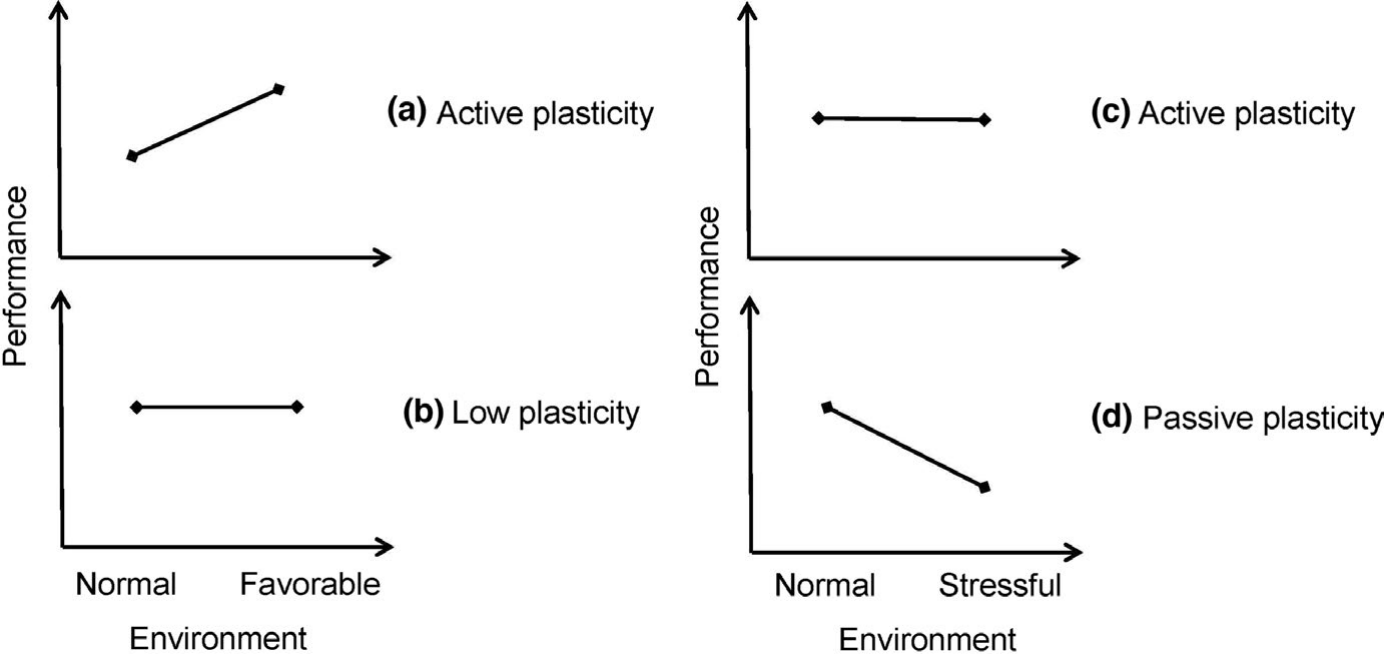
Hypothesized performance in a given trait in response to favorable (a, b) and stressful (c, d) environments, showing active plasticity (a, c), low plasticity (b), and passive plasticity (d)

Phenotypic plasticity in traits is not always adaptive (Bradshaw, 1965; Ghalambor et al., 2007; van Kleunen & Fischer, 2005; Schlichting, 1986; Steams, 1989). The increases (positive/active plasticity) and decreases (negative/passive plasticity) in traits can be regarded as adaptive and nonadaptive/maladaptive, respectively. In response to more favorable conditions, plants may have improved or stable performances in traits, displaying active or low plasticity (Figure 5a, b); in response to more‐stressful environments, no change or decrease in trait performance is apparently regarded as low plasticity or passive plasticity (Figure 5c, d). However, low plasticity may actually manifest greater adaptability than passive plasticity in response to stress and should be considered as adaptive plasticity as active plasticity (Figure 5a, c). An absence of response to environment does not necessarily mean that a plant lacks plasticity (Schlichting, 1986), we should understand phenotypic plasticity as a kind of capability to make adjustments, which might be physiological and invisible, regardless of stable or variable final phenotypes. In this sense, plasticity is a process resembling developmental canalization, as they both function as the mechanism of buffering against environmental disturbances (Waddington, 1952, 1956), sometimes minimizing phenotypic variations, sometimes enlarging such variations. This is especially true when we also believe that decreased canalization can either be advantageous or disadvantageous, depending on specific circumstances.

### 4.4 Correlations between canalization and plasticity

Correlations between plasticity and canalization were not significant in most cases, suggesting the complexity of their relationships, including both positive and negative associations. This may explain why little evidence exists for the correlations between canalization and plasticity (Debat et al., 2009; Dworkin, 2005), despite they are hypothesized to be controlled under some common mechanisms (Debat & David, 2001; Meiklejohn & Hartl, 2002). Nevertheless, we found a few cases of positive correlations between absolute plasticity and canalization, for example, in fertile soil at 50 days and in infertile soil at 70 days, for plants at low and medium densities, respectively. These suggested some shared mechanisms of the two processes in response to environmental stress (Debat & David, 2001; Meiklejohn & Hartl, 2002), and positive correlation is more likely to occur under relatively less stressful or more favorable environments. This probably because that lower level of canalization is not always detrimental. It is reported that plasticity and within‐environment variation are favored by environmental stresses in the unpredictable environments of Mediterranean ecosystem (Valladares et al., 2002). The increase in among‐individual variation in traits may either reflect the potential of traits to react to more favorable environment, or a failure to adapt to environmental stress. In the former case, decreased canalization should be advantageous for active plastic responses, leading to negative correlations between canalization and plasticity; in the latter case, both decreased canalization and passive plasticity may reflect the inability of plants to cope with adverse environmental effects, thus positive correlations between canalization and plasticity may increase.

Increased density, as a biotic stress, can result in greater size variation among individuals, due to the increase of small plants resulting from asymmetry competition in a dense population (Wang, 2006; Weiner, 1985). Both passive plasticity and decreased canalization should be an inevitable outcome in dealing with increased density. In this scenario, greater canalization should be more beneficial for reducing the degree of decrease in traits, thus positive correlations between canalization and plasticity may increase, counteracting negative correlations, leading to insignificant relationships between them. In this sense, canalization can be advantageous or disadvantageous, depending on environmental conditions. In favorable conditions, decreased canalization or greater among‐individual variations may give rise to greater potential to self‐adjust and active plastic responses. In stressful environments, decreased canalization reflects adverse environmental effects, similarly as passive plasticity, increased canalization is needed for stabilizing phenotypes. The environment dependence of the relationship between canalization and plasticity may at least partly explain the mixed results in relevant studies.

### 4.5 Correlations between integration and plasticity

Different from the correlation between canalization and plasticity, phenotypic integration positively correlated with the magnitude of plasticity across different densities, suggesting that traits of greater plasticity were more likely to have higher level of phenotypic integration, or vice versa. Buffering against stresses might be a consequence of developmental complexity rather than simply an evolved resistance mechanism for resilience to stressors (Meiklejohn & Hartl, 2002; Rice, 1998; Siegal & Bergman, 2002; Waxman & Peck, 1998). Under this scenario, the complexes of traits that share the greatest number of developmental interactions (i.e., the most developmentally integrated) should be the most able to maintain functionality and to accommodate the effects of stress during ontogeny. Integration can alleviate the constraints to trait plasticity by environmental signal amplification or inhibition through developmental interaction among trait plasticity (Lande, 2019).

However, if we regard the lesser decrease or lower degree of passive response in traits as an adaptive plasticity (Figure 5c), it is also reasonable to argue that lower level of integration is facilitative for plasticity. Greater flexibility of individual systems is hypothesized to be produced by lessening their homeostatic integration (West‐Eberhard, 2003). The decrease in phenotypic integration might enhance the range of performance of individual organismal systems and ultimately increase organismal capacity to adapt to changing conditions (Badyaev et al., 2005; Rutherford, 2003). The controversy in this issue may derive from different perspectives under different environmental contexts. In this study, however, we found that no matter the passive plasticity in response to high/medium versus low density, or active plasticity in response to low versus medium/high density, greater degree of plasticity always correlated with increased correlations among traits and higher level of integration. It is possible that phenotypic integration is merely an outcome of the plastic responses, rather than a mechanism constraining or facilitating plasticity, thus regardless of the presence of environmental stress, active or passive response in traits, plasticity should most probably lead to increased phenotypic integration.

### 4.6 Effects of soil conditions and growth stage

Our results showed responses of canalization and integration to density mainly occurred at 50 days, but not at 70 days; and correlations among integration, canalization, and plasticity increased over time in infertile soil and decreased over time in fertile soil, consistent with the changes in the morphological responses to density with soil conditions and growth stages (Wang et al., 2017). In a dense population, the strength of among‐individual competition increases with lower availability of belowground resources; and it first increases then decreases as plants grow, and the final decrease may be due to small individuals being obsoleted in the process of “self‐thinning.” Soil condition and growth stage may affect phenotypic integration and canalization as well as their correlations with plasticity through modifying plant size and competition intensity.

## 5 CONCLUSIONS

Our results showed correlations between canalization and plasticity were insignificant in most cases, with a few positive ones between canalization and absolute plasticity at low and medium densities, suggesting the complexity of their relationships and that positive correlations are more likely to occur under more favorable conditions. These revealed that decrease in canalization can be either advantageous or disadvantageous, depending on specific environments. In benign environment, decreased canalization may indicate greater potential to self‐adjust and facilitate active plastic responses. In stressful environment, decreased canalization reflects adverse environmental effects, similarly as passive plasticity, higher canalization should be more beneficial for maintaining trait performance, thereby positive correlations between canalization and plasticity may increase and counteract their negative correlations, leading to no significant correlative results. By contrast, positive correlations between phenotypic integration and plasticity across different densities suggested that it may not be that correlations among traits constrain the evolution of trait plasticity, but that higher level of integration is the outcome of plastic responses.

Responses to density in correlations between integration, canalization, and plasticity became stronger over time in infertile soil but weaker over time in fertile soil, suggesting soil conditions and growth stage may affect responses of these correlations via effects on plant size, competition intensity, and plastic responses in traits. Passive plastic responses, decreased canalization, and increased integration in traits with higher densities revealed negative effects of increased density. The lack of response to stress may demonstrate a stronger ability of adaptation as active response, compared to passive response, thus should be considered as adaptive plasticity. Plants are able to modify the expression and extents of phenotypic plasticity, integration, and canalization in traits as well as their relationships, depending on different environmental contexts, to better adaptation in the changing world. Exactly by virtue of such flexibility in regulating different processes and their relationships, plant species can maintain appropriate magnitudes of expression in each of them in the long history of evolution.

## CONFLICT OF INTEREST

The authors have no conflict of interest to declare.

## AUTHOR CONTRIBUTION

**Shu Wang:** Conceptualization (lead); Project administration (lead); Data curation (lead); Formal analysis (lead); Funding acquisition (lead); Investigation (lead); Methodology (lead); Visualization (lead); Writing‐original draft (lead); Writing‐review & editing (lead). **Dao‐Wei Zhou:** Conceptualization (supporting); Funding acquisition (supporting); Methodology (supporting).

## Data Availability

Data are available via the Dryad Digital Repository: http://doi.org/10.5061/dryad.bvq83bk8c

## APPENDIX 1

**TABLE A1:** *F*‐values for three‐way ANOVA or ANCOVA on all traits, with growth stage (GS), soil condition (SC), population density (PD), and their interactions as effects, with log_10_(TM) nested within stage as a covariate in ANCOVA

Trait	TM (*df* = 1)	GS (*df* = 2)	SC (*df* = 1)	PD (*df* = 2)	GS × SC (*df* = 2)	GS × PD (*df* = 4)	SC × PD (*df* = 2)	SC × GS × PD (*df* = 4)
For three stages								
TM		2,601.19***	121.88***	60.54***	42.40***	7.71***	7.15***	3.11*
RMR	2.21	32.51***	8.26**	1.04	3.09*	2.19	1.90	1.41
SMR	9.71***	594.71***	0.01	17.64***	72.31***	4.88***	0.25	2.63*
LMR	4.95**	516.42***	54.44***	1.19	63.91***	2.82*	2.08	0.63
SL	144.89***	4,782.12***	333.33***	0.72	44.73***	5.92***	1.70	5.20***
*SD*	61.30***	987.83***	60.61***	47.29***	7.02**	2.13	6.99**	0.38
PA	16.16***	155.88***	8.69**	13.71***	2.84	1.86	5.62**	2.98*
LN	42.56***	192.95***	256.16***	39.82***	7.56**	4.47***	2.33	2.95*
LS	142.26***	3,214.65***	129.41***	74.37***	108.27***	1.91	3.93*	0.62
For the 2nd and 3rd stages								
TM		132.09***	166.00***	62.11***	0.98	0.18	8.20***	2.88
PMR	13.40***	294.00***	25.19***	0.32	68.99***	1.62	2.45	0.09
LAMR	1.60	146.78***	110.44***	17.61***	58.55***	0.67	0.38	0.66
REMR	2.51	233.34***	101.43***	2.16	6.27*	0.42	2.23	1.11
MRL	5.51**	54.37***	1.50	9.03***	16.14***	3.03*	0.81	1.44
MRD	131.22***	86.52***	374.02***	135.81***	11.91***	2.24	0.61	2.95
LRL	0.73	57.36***	19.03***	74.13***	61.99***	1.88	0.11	2.17
LRN	38.97***	7.04**	106.81***	42.70***	2.09	0.38	2.89	3.44*
PL	43.29***	286.01***	546.52***	82.10***	39.58***	2.02	1.82	2.81
For the 3rd stage only								
TM			49.29***	12.28***			4.83*	
BMR	14.86***		74.21***	6.55**			1.51	
BL	93.30***		7.85**	5.99**			6.52**	
BA	10.45**		19.68***	3.32*			3.31*	
BN	76.37***		5.81*	6.47**			0.17	

Note

**p* < .05, ***p* < .01, ****p* < .001.

**TABLE A2:** The number of significant correlations for each trait with other traits (NC) at low (L), medium (M), and high (H) densities under infertile and fertile soil conditions at 50 and 70 days of plant growth

Trait	50 days						70 days					
	Infertile			Fertile			Infertile			Fertile		
	L	M	H	L	M	H	L	M	H	L	M	H
TM	5	8	7	10	9	10	9	7	10	7	5	7
RMR	5	5	2	5	5	5	3	2	6	0	5	2
SMR	6	2	2	5	5	2	4	4	4	3	2	2
PMR	8	2	2	6	3	3	5	4	4	2	5	5
LAMR	5	2	3	2	4	5	4	4	3	6	3	2
REMR	1	2	2	3	3	2	1	4	1	6	4	3
SL	1	6	2	5	5	3	4	3	4	0	2	2
*SD*	1	1	1	2	7	0	3	3	0	0	4	4
MRL	5	5	0	2	1	4	2	1	2	0	0	2
MRD	3	4	0	5	7	2	1	4	3	7	3	3
LRL	3	2	3	1	2	3	2	3	4	0	1	1
LRN	2	0	0	1	4	0	3	2	0	3	2	2
PL	4	4	4	7	7	4	2	2	5	6	4	2
PA	0	0	3	1	1	3	2	1	1	1	1	1
LN	4	3	4	2	5	1	5	1	2	5	1	3
LS	1	3	2	3	5	3	1	8	1	5	7	4

Note

Abbreviations for all traits and variables are in Table 1.

**TABLE A3:** Coefficient of variation (CV) for each trait at low (L), medium (M), and high (H) densities under infertile and fertile soil conditions at 50 and 70 days of plant growth

Trait	50 days						70 days					
	Infertile			Fertile			Infertile			Fertile		
	L	M	H	L	M	H	L	M	H	L	M	H
TM	**0.357**	**0.400**	**0.416**	0.199	**0.421**	**0.503**	**0.532**	**0.336**	**0.449**	0.039	0.083	**0.395**
RMR	0.123	0.262	**0.380**	0.164	0.274	0.225	**0.324**	**0.505**	**0.323**	**0.344**	0.255	0.201
SMR	0.118	0.112	0.154	0.099	0.177	0.088	0.197	0.248	**0.309**	0.148	0.112	0.142
PMR	0.114	0.260	0.277	0.155	0.120	0.131	**0.454**	**0.300**	0.276	0.219	0.182	0.236
LAMR	0.094	0.170	0.120	0.065	0.131	0.138	**0.521**	**0.306**	0.252	0.187	0.172	0.141
REMR	**0.502**	**0.923**	**1.025**	**0.526**	**0.465**	**0.674**	**0.641**	**0.352**	0.299	**0.482**	**0.738**	**0.605**
SL	0.264	0.176	0.235	0.200	0.215	0.204	0.296	0.160	0.264	0.129	0.184	0.129
*SD*	0.222	0.159	0.143	0.211	0.206	**0.303**	0.164	0.067	0.222	0.196	0.164	1.158
MRL	0.283	0.283	**0.448**	**0.361**	0.239	**0.399**	0.183	0.183	0.133	**1.077**	0.154	0.360
MRD	0.143	0.169	0.141	0.191	0.208	**0.329**	0.183	0.118	0.201	0.189	0.176	**0.341**
LRL	0.230	0.178	0.296	0.178	0.273	**0.448**	0.150	0.229	0.226	0.220	0.168	0.165
LRN	**0.317**	**0.401**	0.289	0.295	**0.490**	**0.576**	**0.374**	**0.352**	**0.519**	0.252	**0.305**	**0.467**
PL	0.166	0.222	0.224	0.135	0.172	**0.314**	0.150	0.130	0.168	0.126	**0.452**	**0.609**
PA	0.157	0.170	0.216	0.186	0.138	0.232	0.120	0.113	0.088	0.103	0.147	0.146
LN	0.123	0.184	0.228	0.135	0.108	0.197	0.171	0.211	0.159	0.147	0.123	0.247
LS	0.163	0.125	0.155	0.146	0.128	0.193	0.162	0.113	0.135	0.144	0.136	0.212

Note

Abbreviations for all traits and variables are in Table 1. Bold fonts indicate significant CV values (*p* < .05).

**TABLE A4:** Relative plasticity (PI_rel_) in response to high versus low density (H‐L) and medium versus low density (M‐L) for each trait under infertile and fertile soil conditions at 50 and 70 days of plant growth

Trait	50 days				70 days			
	Infertile		Fertile		Infertile		Fertile	
	H‐L	M‐L	H‐L	M‐L	H‐L	M‐L	H‐L	M‐L
TM	**−0.474**	−0.034	**−0.438**	**−0.336**	**−0.292**	−0.006	**−0.528**	**−0.393**
RMR	**−0.548**	0.077	−0.059	0.004	−0.074	0.047	0.014	−0.026
SMR	**0.209**	**0.073**	**0.142**	**0.078**	**0.098**	**0.149**	0.055	0.017
PMR	−0.132	−0.017	**−0.149**	**−0.072**	**−0.365**	−0.109	−0.021	0.030
LAMR	0.035	**−0.058**	**−0.065**	−0.040	**−0.368**	**−0.125**	−0.010	0.002
REMR	−0.051	0.061	−0.190	−0.199	0.183	−0.003	−0.029	−0.023
SL	**0.164**	0.057	**0.168**	0.050	**0.129**	**0.120**	0.007	−0.045
*SD*	−0.016	−0.015	0.024	0.013	0.014	0.021	−0.081	−0.014
MRL	0.057	0.108	0.111	0.082	−0.014	0.042	0.069	−0.082
MRD	**−0.065**	−0.053	0.032	0.006	**−0.059**	−0.020	−0.050	−0.025
LRL	**−0.352**	−0.110	**−0.215**	**−0.135**	**−0.221**	**−0.090**	**−0.208**	−0.014
LRN	**−0.388**	−0.033	0.065	0.108	−0.031	0.183	−0.167	−0.133
PL	0.005	−0.014	**−0.035**	−0.017	**−0.080**	−0.009	**−0.228**	−0.037
PA	**−0.176**	−0.036	−0.009	0.033	−0.015	**−0.056**	0.007	0.033
LN	**−0.170**	−0.049	−0.014	−0.038	**−0.079**	0.002	−0.030	−0.014
LS	0.096	**0.075**	0.006	−0.009	**−0.085**	−0.024	−0.043	−0.017

Note

The values in bold font indicate significant PIs. Abbreviations for all traits and variables are in Table 1.
